# Impact of *in-utero* exposure to HIV and latent TB on infant humoral responses

**DOI:** 10.3389/fimmu.2024.1423435

**Published:** 2024-06-27

**Authors:** Kimberly J. S. Hjelmar, Lesley R. de Armas, Evan Goldberg, Suresh Pallikkuth, Jyoti Mathad, Grace Montepiedra, Amita Gupta, Savita Pahwa

**Affiliations:** ^1^ Department of Microbiology and Immunology, University of Miami Miller School of Medicine, Miami, FL, United States; ^2^ Center for Biostatistics in AIDS Research, Harvard T.H. Chan School of Public Health, Boston, MA, United States; ^3^ Department of Medicine, Department of Obstetrics & Gynecology, Weill Cornell Medical College, New York City, NY, United States; ^4^ Division of Infectious Diseases, Johns Hopkins University School of Medicine, Baltimore, MD, United States

**Keywords:** TB, HIV, infants, HEU, antibodies, BCG, pregnancy

## Abstract

**Introduction:**

Latent tuberculosis infection (LTBI) is a common coinfection in people living with HIV (PWH). How LTBI and HIV exposure in utero influence the development of infant humoral immunity is not well characterized. To address this question, we assessed the relationship between maternal humoral responses in pregnant women with HIV or with HIV/LTBI on humoral responses in infants to BCG vaccination and TB acquisition.

**Methods:**

Plasma samples were obtained from mother infant pairs during pregnancy (14-34 wks gestation) and in infants at 12 and 44 wks of age from the IMPAACT P1078 clinical trial. LTBI was established by Interferon gamma release assay (IGRA). Progression to active TB (ATB) disease was observed in 5 women at various times after giving birth. All infants were BCG vaccinated at birth and tested for IGRA at 44 weeks. Mtb (PPD, ESAT6/CFP10, Ag85A, LAM), HIV (GP120), and Influenza (HA) specific IgG, IgM, and IgA were measured in plasma samples using a bead based Luminex assay with Flexmap 3D.

**Results:**

In maternal plasma there were no differences in Mtb-specific antibodies or viral antibodies in relation to maternal IGRA status. ATB progressors showed increases in Mtb-specific antibodies at diagnosis compared to study entry. However, when compared to the non-progressors at entry, progressors had higher levels of Ag85A IgG and reduced ESAT6/CFP10 IgG and LAM IgG, IgM, and IgA1. All infants showed a decrease in IgG to viral antigens (HIV GP120 and HA) from 12 to 44 weeks attributed to waning of maternally transferred antibody titers. However, Mtb-specific (PPD, ESAT6/CFP10, Ag85A, and LAM) IgG and IgM increased from 12 to 44 weeks. HIV and HA IgG levels in maternal and 12-week infant plasma were highly correlated, and ESAT6/CFP10 IgG and LAM IgG showed a relationship between maternal and infant Abs. Finally, in the subset of infants that tested IGRA positive at 44 weeks, we observed a trend for lower LAM IgM compared to IGRA- infants at 44 weeks.

**Discussion:**

The results from our study raise the possibility that antibodies to LAM are associated with protection from progression to ATB and support further research into the development of humoral immunity against TB through infection or vaccination.

## Introduction

*Mycobacterium tuberculosis* (*Mtb*) is a major cause of human disease worldwide. In 2023, there were an estimated 10.6 million cases of active tuberculosis (ATB) and 1.3 million TB-related deaths. Women accounted for 3.5 million cases of TB while children accounted for 1.3 million (1). Studies have shown that women are at increased risk for developing ATB disease during pregnancy and post-partum (2, 3). *Mtb* infection during pregnancy has been shown to result in poor outcomes in infants including fetal distress, low birth weight, prematurity, and infant mortality (4, 5). Furthermore, countries where TB is endemic often have a strong incidence of HIV as well, with TB being a leading cause of death in people living with HIV (PLWH) (6, 7). HIV infection has contributed to poor infant outcomes including low birth weight, preterm birth, and small size for gestational age, with and without the use of ART (8, 9). Exposure to HIV *in utero* has also been shown to create immunological challenges for infants with and without infection with HIV. Studies have shown that HIV-exposed uninfected (HEU) infants exhibit altered immunological profiles compared to unexposed infants including decreased lymphocyte counts increased cellular activation (10, 11), presence of HIV-specific T cell responses (12–14), and poor cellular responses to vaccination with Bacille Calmette-Guerin (BCG) (15, 16). The impact of *in utero* exposure to both HIV and TB on the infant immune system has not been studied in depth. Some studies have suggested that infants may have transient changes in their immune response to PPD, ESAT6/CFP10, or BCG but they concluded these changes were unlikely to impact infant responses to BCG (17, 18).

Maternal antibodies are transferred to infants during pregnancy via the neonatal Fc receptor. More recent literature has shown a role for TB-specific antibodies and their Fc-receptor based functional profiles in the context of TB immunity and protection (19). Therefore, understanding the profile and function of circulating antibodies in infants remains an untapped area of study (20, 21). In women living with HIV, Fc reactivity of HIV-specific antibodies were reduced in women who transferred HIV to their infants. Furthermore, infants who did not become HIV infected had greater neonatal Fc receptor (FcRn) reactivity resulting in greater transfer of HIV-specific maternal IgG (22). In the current study, we evaluated the HIV- and *Mtb*- specific antibody profiles of mother/infant pairs using a bead-based Luminex assay to determine (1) if antibody profiles in mothers and infants differed based on maternal LTBI status and (2) if antibody profiles were associated with the infants’ protection from TB acquisition in the first year of life.

## Methods

### Study population

All participants in the current study were enrolled in the IMPAACT P1078/TB APPRISE TB-prevention trial which assessed the safety of initiating isoniazid treatment (IPT) during pregnancy versus after delivery in women living with HIV from high TB incidence settings (23). 956 women were enrolled from eight countries (Tanzania, South Africa, Botswana, Haiti, Zimbabwe, India, Thailand, Uganda) during pregnancy; mother-infant pairs were followed for 48 weeks post-partum. A subset of enrolled mother-infant pairs (n=286) gave plasma and PBMCs at study entry (maternal; 14–34 weeks gestation) and 12 and 44 weeks of age (infant). Most infants received BCG vaccination within 48 hours after birth. TBI was assessed at study entry in mothers and infants at 44 weeks using the QuantiFERON-TB Gold In-Tube (QGIT), an IFN gamma release assay (IGRA). PBMC and plasma were isolated from blood samples and stored onsite or shipped to IMPAACT sample repository (BRI) for storage prior to shipping to University of Miami for the current study. 98 IGRA- and 100 IGRA+ mothers were included. 5 women were diagnosed with possible, probable, or confirmed TB as determined by the site. Infants with plasma available at both 12 and 44 weeks were included for analysis of infant data. The characteristics for the subset of TB Apprise cohort participants included in this study are shown in **Table 1**.

**Table 1:** Maternal participant characteristics.

	IGRA-	IGRA+	P-Value
**n**	98	100	
**Age, years (median, IQR)**	18–44 (30, 9)	18–44 (30, 10)	0.8407
**Gestational Age, weeks (median, IQR)**	14–34 (26, 8)	14–34 (27, 8)	0.7158
**CD4 cells/mm^3^ (median, IQR)**	143–1047 (467, 355)	58–1411 (494, 226)	0.6451
**Plasma Virus Load (median, IQR)**	39–464894 (39, 88)	19–60475 (39, 61.25)	0.6709
**INH Arm (Immediate)**	51	49	
** INH Arm (deferred)**	47	51	
**Infants** (n)	87	83	
**IGRA+ Infants** (n)	3	6	
**Age at BCG, weeks (median)**	0–65 (1)	0–69 (1)	
**Infant Weight, grams (median, IQR)**	2000–4200 (2950, 617.5)	1550–4020 (3120, 501.25)	0.3377

IQR, Interquartile Range; INH, Isoniazid; IGRA, Interferon gamma release assay; BCG, Bacille-Calmette Guerin Vaccine.

### Quantification of antigen-specific Ig isotypes

#### Bead coupling of protein antigens

Magplex-C Microspheres (Luminex) were coupled to protein antigens of interest as previously described (24). Antigens included were HIV GP120 (HIV-1/Clade B/C Consensus)(Immune Tech), *Mycobacterium tuberculosis* PPD (AJ Vaccines), *Mycobacterium tuberculosis* ESAT6 (BEI Resources, *Mycobacterium tuberculosis* CFP10 (BEI Resources), *Mycobacterium tuberculosis* Ag85A (BEI Resources), Recombinant Zaire Ebola Virus GPΔTM (IBT Bioservices), and Influenza HA (H1N1/A/New Caledonia/20/99, H1N1/A/Solomon Islands/3/2006, and H3N2)(A/Brisbane/10/2007) (Immune Tech). Briefly, 5 million Magplex-C Microspheres were washed using 100 µL dH_2_0 by vortexing for 30 seconds. Following washing, beads were resuspended in 80 µL activation buffer (3g NaH_2_PO_4_, 40 drops 5N NaOH in 250 mL dH_2_0). 10 µL of 50 mg/mL Sulfo-NHS and 50 mg/mL EDC were added to the microspheres and mixed via vortex. Microspheres were incubated for 30 minutes at room temperature on a rotator. Following incubation, microspheres were washed 3 times with 250 µL of coupling buffer (2.44 g MES, 5 drops 5N NaOH in 250 mL dH_2_0). After washing, 25 µg of non-biotinylated protein and a total volume of 500 µL of coupling buffer was added and mixed via vortex. This was followed by a 2-hour incubation by rotation at room temperature. Microspheres were then resuspended in 500 µL of blocking buffer, mixed by vortex, and incubated for an additional 30 minutes by rotation at room temperature. After blocking, microspheres were washed 3 times with 1 mL of wash buffer (500 µL Tween-20 in 1 L 1X PBS). The coupled microspheres were then resuspended in 250 µL PBS.

#### Bead coupling of glycolipid antigens (LAM)

Prior to bead coupling, 4-(4,6-Dimethoxy-1,3,5-triazin-2-yl)-4-methylmorpholinium chloride (DMTMM) (Thermofisher) was freshly diluted to 200 mg/mL by adding 50 µL of ACS-grade H_2_0 to 10 mg of stock solution. ACS-grade H_2_0 was used throughout this assay. 2 µL of diluted DMTMM was added per 25 µg of LAM (BEI Resources). More H_2_0 was added to bring the total volume to 300 µL. The DMTMM and antigen solution was incubated for 1 hour at room temperature. During this incubation, columns (Cytiva) were equilibrated with H_2_0. Equilibration began by removing the inner filter and cutting the end to allow the storage solution to flow out. The column was placed in a 50 mL tube, filled with 2.6 mL of H_2_O, centrifuged at 1000 x g for 2 minutes and flow through was discarded. This step was repeated 3 times. Following equilibration, 300 µL of DMTMM/Antigen solution was added to the column and allowed to enter completely before adding 400 µL of H_2_O and centrifuging at 1000 x g for 2 mins. The flow through was discarded. 500 µL of H_2_0 was added and the eluate collected. 400 µL of microspheres were washed with 200 µL of H_2_O and resuspended in 40 µL of water. 40 µL of microspheres were added to 500 µL of DMTMM-polysaccharide solution and incubated overnight at room temperature. Microspheres were washed 4 times with 500 µL of PBS and resuspended in 375 µL of PBS.

#### Luminex assay

Maternal and infant plasma was diluted at a ratio of 1:200 and 5 µL of diluted plasma was added per well of a 96 well, PS, F-bottom, medium binding, microplate in black (Greiner Bio-one), centrifuged at 112 x g for 1 minute and covered with an ELISA plate cover. Each sample was run in duplicate. Antigen-coupled microspheres were added at 30 µL (~6x10^5^ beads) per bead region to 18 mL of Luminex assay buffer (0.5 mL Tween-20, 1 g BSA in 1 L 1X PBS) and vortexed. 45 µL of bead solution was then added to each well and covered with a foil plate cover. Plates were incubated on a shaker overnight at 4°C. The following day, the plate was spun down at 112 x g for 1 minute and washed 3 times with 100 µL of Luminex assay buffer using a Biotek ELx50 96 well plate washer. Antibodies were prepared by adding 26 µL of secondary antibody to 8 mL of Luminex assay buffer. Secondary antibodies include mouse anti-human IgG-PE (Southern Biotech), mouse anti-human IgM-PE (Southern Biotech), and mouse anti-human IgA1-PE (Southern Biotech). 40 µL of the diluted secondary antibody was added per well and covered. Plates were incubated on a shaker for 1 hr at room temperature and followed with washing 3 times in 100 µL of Luminex assay buffer. Microspheres were resuspended in 100 µL of wash buffer and run in duplicates on a Luminex Flexmap 3D Instrument. Results were reported as mean fluorescence intensity (MFI) of the stained antibody. Following data collection, quality control was run to eliminate samples that had collected fewer than 20 beads per region. The means of the duplicates of the MFI per antigen was calculated and used for downstream analysis.

### Statistical analysis

Mann-Whitney test was used to evaluate significant differences (p<0.05) in the MFI of HIV-, *Mtb*-, and HA-specific IgG, IgM, and IgA1 in IGRA- and IGRA+ mothers. This was also used to compare the main cohort, or women who did not progress to ATB, at entry to ATB mothers at entry. Wilcoxon Sign Rank test was used to compare HIV, HA, and TB-specific antibody responses from entry to diagnosis among mothers who developed ATB. Following this analysis, mothers were classified into antibody (Ab) positive and Ab negative responses. Two times the mean of the PBS control plus the SD was used to distinguish Ab positive from Ab negative responses. IgA1 was excluded from classified analysis due to insufficient values for analysis. Fisher’s exact test was used to evaluate significant differences (p<0.05) in antibody positivity between the main cohort and women who progressed to ATB. Spearman correlation was used to evaluate correlations (p<0.05) between classified maternal *Mtb*-, HIV-, and HA- IgG, IgM, and IgA with maternal clinical parameters (age, CD4 count, viral load, gestational age, infant weight, and quantitative IGRA at entry, delivery, and postpartum). Mann-Whitney test was also used to identify significant differences in antibody responses (p<0.05) between infants from IGRA- and IGRA+ mothers. Infants were further evaluated using the Wilcoxon Sign Rank test to evaluate significant (p<0.05) differences in HIV-, *Mtb*-, and HA- IgG, IgM, and IgA1 from weeks 12 to 44. Benjamini-Hochberg was performed with family-wise error rate (FWER) of 5% to correct for multiple testing in infants. Fisher’s exact test was used to evaluate significant differences (p<0.05) in antibody positivity in infants from 12 to 44 weeks. Infant antibody responses were classified into positive and negative responses in the same manner as maternal data. To get a better understanding of the relationship between maternal IgG levels during pregnancy and the IgG levels in infants, Mann-Whitney U test was used to assess significant changes in maternal antibody responses based on infant antibody positive or antibody negative responses to *Mtb* antigens. Linear regression was also used to evaluate significant associations between maternal and infants IgG (12 weeks) to viral antigens (HIV and HA). IGRA+ infants were also classified as described and Mann-Whitney test was used to identify significant differences (p<0.05) in HIV-, *Mtb*-, and HA- IgG and IgM between IGRA- and IGRA+ infants and between IGRA+ and IGRA- mothers within IGRA+ infants.

### Outcome measures

We measured IgG, IgM, and IgA1 antibody levels by MFI specific for 4 common *Mtb* antigens (PPD, ESAT6/CFP10, Ag85A, and LAM) in maternal plasma during the 2^nd^ or 3^rd^ trimester. We also measured antibodies to viral antigens, Influenza hemagglutinin (HA) and HIV-1 envelope (GP120) as a control for maternal transfer of Abs. These measures were repeated in infant plasma.

## Results

### IGRA status does not impact clinical parameters in mothers

We first sought to evaluate differences in maternal clinical parameters based on IGRA status. All women included in this study were HIV positive and classified as IGRA+ or IGRA- based on an IGRA test taken at the time of enrollment. No significant differences were observed in maternal clinical characteristics between IGRA+ and IGRA- groups, including age at study entry which ranged from 18–44 years in both groups with a median of 30 years (**Table 1**). Parameters associated with HIV were also assessed including CD4 count and plasma viral load. The median CD4 count for IGRA- mothers was 467 with an IQR of 355 while the median CD4 count in IGRA+ mothers was 494 with an IQR of 226. 79.59% of IGRA- women and 82% of IGRA+ women had a viral load below 200 copies/mL. Median viral load for IGRA- non suppressors was 511.5 with an IQR of 1610. The median viral load of IGRA+ non suppressors was 1776.5 with an IQR of 8060.5. The participants were matched for INH arm with 51 IGRA- and 49 IGRA+ women from the immediate INH arm and 47 IGRA- and 51 IGRA+ women from the deferred INH group. At 44 weeks of age, 3 infants from IGRA- mothers and 6 infants from IGRA+ mothers tested IGRA positive. 5 women in the substudy cohort progressed to ATB over the course of the study, 2 from the IGRA- group, 2 from the IGRA+ group, and 1 with an intermediate status; samples obtained at the time of ATB diagnosis were included in this study.

### *Mtb*-specific antibody responses in maternal plasma during pregnancy are dominated by LAM IgG and Ag85A IgM regardless of IGRA status

**Figure 1:**
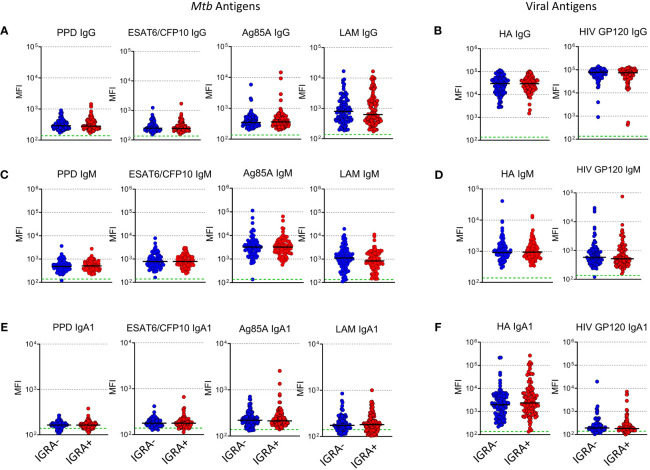
Levels of plasma *Mtb*-specific antibody is independent of IGRA status in women from TB-Apprise. Dot plots showing levels of *Mtb* and HIV specific IgG **(A, B)**, IgM **(C, D)**, and IgA1 **(E, F)** in plasma samples from IGRA- women (n=98, blue circles) and IGRA+ (n=100, red circles). Horizontal black lines represent median values for the group. Green dotted line represents the background for the assay (mean PBS control value). Mann-Whitney U test was performed to compared IGRA- and IGRA+ groups, no significant differences were observed.

To evaluate whether LTBI status during pregnancy influences maternal humoral immunity, maternal *Mtb* and HIV specific antibody responses were assessed. Observed median maternal plasma IgG MFI against viral antigens were approximately 1–2 logs higher than those of *Mtb* antigens (**Figures 1A, B**). LAM IgG MFI was highest amongst the *Mtb-* specific IgG suggesting it was the most abundant *Mtb-* specific Ab detected in maternal plasma samples. Ag85A IgM MFI was highest amongst *Mtb*- specific IgM suggesting it was the most abundant of the IgM isotype (**Figures 1C, D**). IgA1 Abs were present at very low levels except for those specific for Influenza HA (**Figures 1E, F**). Ebola- specific IgG was also included as a control and median MFI values were significantly lower than the other antigens tested (**Supplementary Figure 1**). These results indicate that maternal IGRA status does not significantly impact the levels of Abs detected in this cohort.

### Maternal progressors exhibited an increase in *Mtb*-specific antibody responses at ATB diagnosis

**Figure 2:**
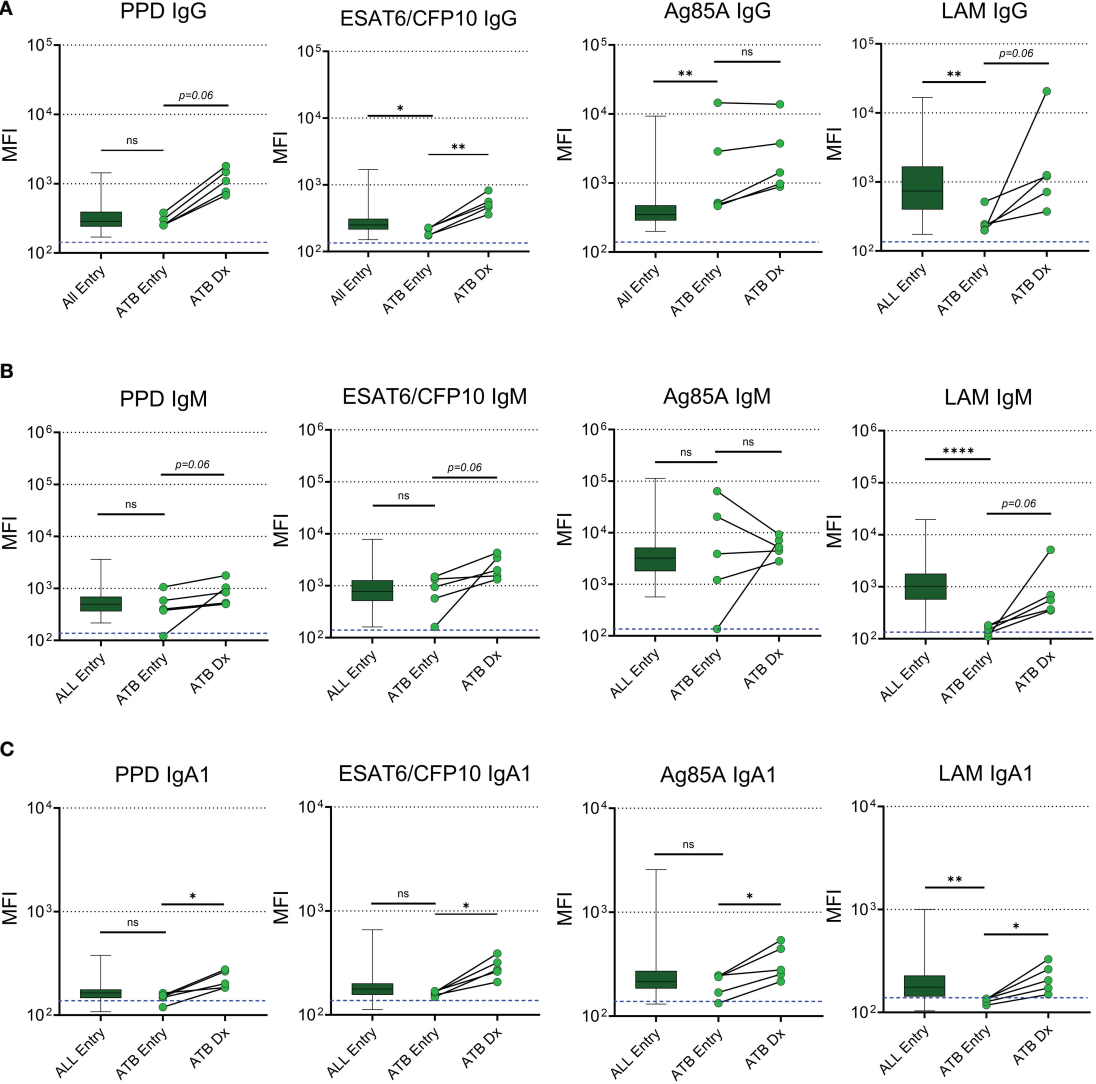
*Mtb*-specific antibodies increase in maternal plasma with active TB diagnosis post-delivery. Dot plots with median comparing levels of TB specific IgG **(A)**, IgM **(B)**, and IgA1 **(C)** in all participants at entry (n=192, excluding active TB progressors) with active TB progressors at study entry and the time of diagnosis (n=5). Blue dotted lines represent the background for the assay (mean PBS control value). Mann-Whitney U test was performed to compare active TB progressors at entry to the main cohort at entry. Active TB progressors were compared from entry to diagnosis using Wilcoxon Sign Rank test. Significant values are represented by asterisks *p ≤ 0.05, **p ≤ 0.01, ***p ≤ 0.001, ****p ≤ 0.0001. ns, stands for non-significant.

A subset of 5 participants progressed to ATB after the entry timepoint and provided blood samples at the time of diagnosis which ranged from 27 to 67 weeks post-entry (**Table 2**). Maternal antibodies were assessed at ATB diagnosis and compared to levels at entry. IgG specific for ESAT6/CFP10 increased during ATB compared to entry and we observed a trend (p=0.06) for increases of IgG against PPD and LAM (**Figure 2A**). Progressors exhibited reduced levels of ESAT6/CFP10 and LAM IgG at entry compared to the main cohort and significantly increased Ag85A IgG relative to the main cohort. Trends were noted for increased IgM antibodies against *Mtb*-specific antigens during ATB with decreased LAM IgM at entry compared to the main cohort (**Figure 2B**). PPD, ESAT6/CFP10, Ag85A, and LAM IgA1 increased from entry to diagnosis. LAM IgA1 was significantly reduced in ATB mothers at entry compared to the main cohort (**Figure 2C**). HIV-, HA-, and Ebola- specific IgG was also assessed and there was no change in MFI from entry to diagnosis in TB progressors (**Supplementary Figure 2**).

**Table 2:** Clinical characteristics of participants that progressed to active TB.

Sample	IGRA	Age (yrs)	CD4 (cells/mm3)	Plasma Virus Load (copies/ml)	Gestational Age (wks)	Infant Birth Weight (grams)	INH Arm	Active TB Diagnosis (wks post-entry)
1	–	40	261	39	31	N/A	Deferred	27
2	–	25	289	39	27	2780	Immediate	38
3	Int	31	79	199	27	3000	Deferred	52
4	+	29	678	39	16	3127	Immediate	34
5	+	21	540	39	21	3525	Immediate	67

### LAM-specific antibodies are reduced in maternal ATB progressors at entry

**Figure 3:**
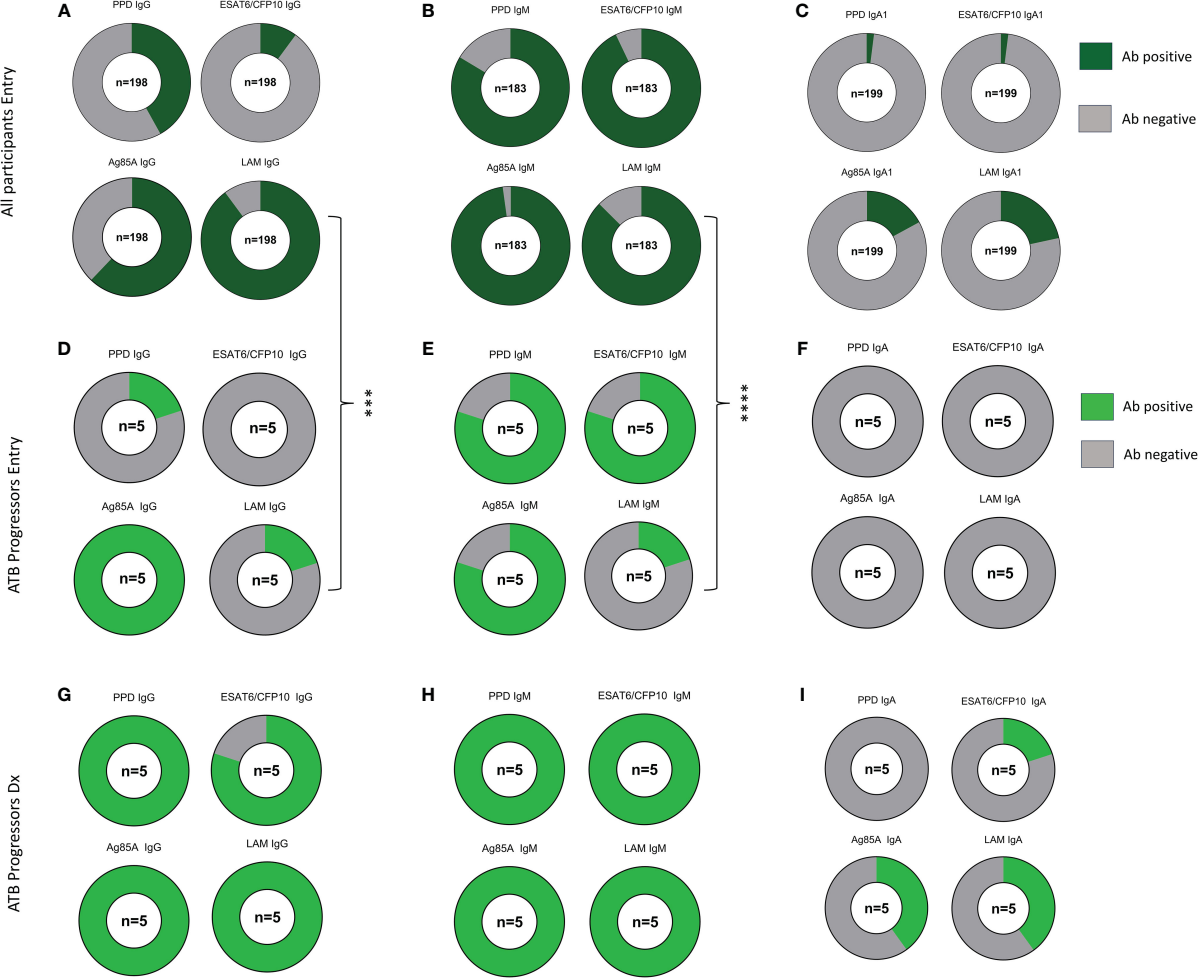
ATB progressors exhibit reduced LAM specific IgG and IgM prior to diagnosis. Part of whole graphs showing classification of IgG, IgM, and IgA1 responses into positive and negative antibody levels in the entire cohort **(A–C)**, ATB progressors at study entry **(D–F)** and at ATB diagnosis **(G–I)**. A fisher’s exact test was used to compare proportion of individuals with positive *Mtb*-specific antibodies between all participants and ATB progressors at study entry. Significant values are represented by asterisks *p ≤ 0.05, **p ≤ 0.01, ***p ≤ 0.001, ****p ≤ 0.0001.

Next, we determined the proportion of maternal samples with positive antibody responses against each of the *Mtb* antigens at study entry using a threshold cutoff of two times the mean + 1SD of the PBS control. IgG to LAM was positive in 89.9% of participants, while IgG against Ag85A, PPD, and ESAT6/CFP10 were present in 62.1%, 41.9%, and 10.1% of participants, respectively (**Figure 3A**). IgM Abs were positive in greater than 80% of participants for all *Mtb* antigens and IgA1 Abs were positive to LAM and Ag85A in 21.6% and 17.1% of participants, respectively (**Figures 3B, C**). This analysis was replicated in ATB progressors both at study entry and at the time of diagnosis and revealed that, at study entry, progressors had a significantly lower chance of having positive LAM IgG and IgM antibody responses compared to the main cohort (**Figures 3D–F**). As shown in the paired analysis, progressors exhibited increased proportions of individuals with positive PPD IgG (p=0.0476), ESAT6/CFP10 IgG (p=0.0476), LAM IgG (p=0.0476), and LAM IgM (p=0.0079) at the time of diagnosis compared to entry (**Figures 3G–I**).

### Maternal *Mtb*-specific antibody levels correlated independent of IGRA status

**Figure 4:**
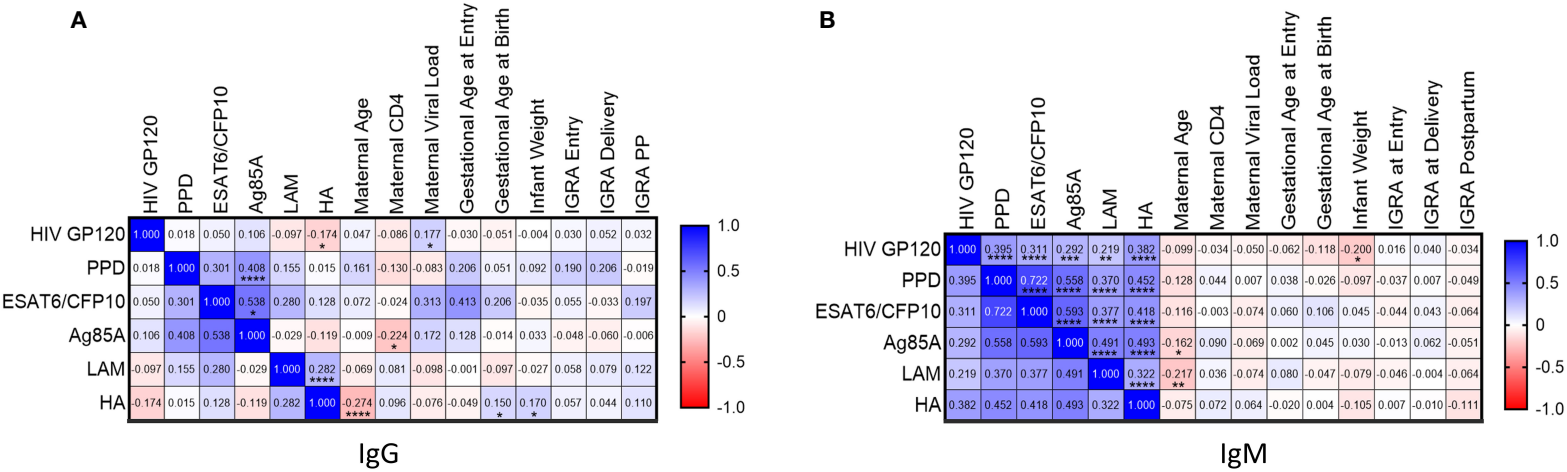
Levels of *Mtb*-specific antibody levels to protein antigens correlate in pregnant women from TB-Apprise Cohort regardless of IGRA status. Spearman correlation of maternal HIV-, TB-, and HA- specific IgG **(A)** and IgM **(B)** antibodies to maternal and infant clinical parameters. Correlation coefficient for each test is printed in the colored box, indicating positive (blue) or negative (red) associations. Significant values are represented by asterisks *p ≤ 0.05, **p ≤ 0.01, ***p ≤ 0.001, ****p ≤ 0.0001.

*Mtb*-specific, HIV-specific, and HA-specific positive Ab measurements were correlated to maternal clinical parameters and quantitative maternal IGRA. Ag85A IgG levels positively correlated with PPD and ESAT6/CFP10 IgG in maternal samples and negatively correlated with CD4 count (**Figure 4A**). LAM IgG on the other hand did not correlate with other *Mtb* antibodies but showed a positive correlation with Influenza HA IgG. HIV GP120 IgG had positive correlation with HIV VL as expected but negatively correlated with IgG to Influenza HA.

All *Mtb*-specific IgM antibodies were positively correlated to each other (**Figure 4B**). Additionally, HIV GP120 IgM and HA IgM were positively correlated with *Mtb*-specific antibodies. Maternal age negatively correlated with Ag85A and LAM IgM. Finally, HIV GP120 IgM negatively correlated with infant weight. Quantitative IGRA results did not show any association with *Mtb*, HIV, or HA specific antibody responses.

### *Mtb*-specific antibodies increase with age in infants independent of maternal IGRA status

**Figure 5:**
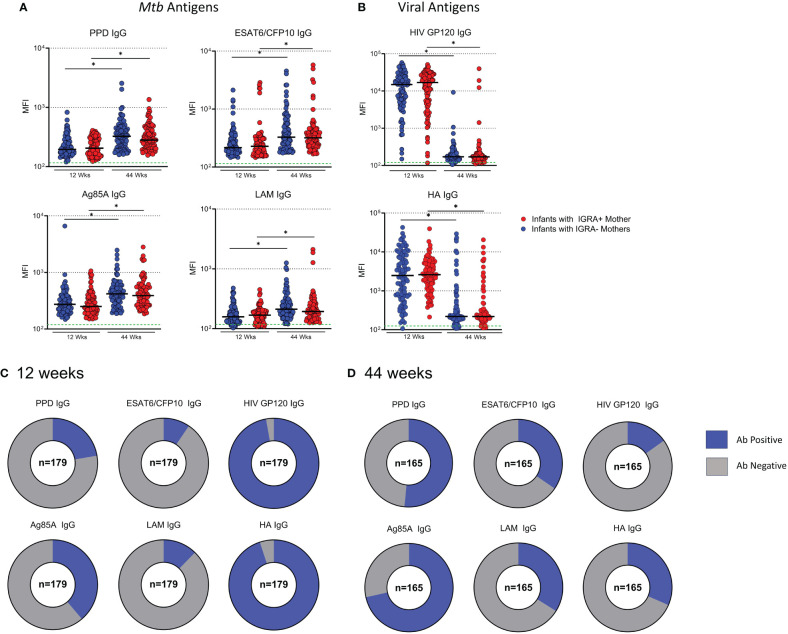
Development of *Mtb*-specific antibodies increase with age in BCG vaccinated infants regardless of maternal IGRA status. Dot plots with median showing a comparison of *Mtb*-specific **(A)** and Virus-specific **(B)** IgG in Infant plasma at 12 and 44 weeks of age. Green dotted lines represent the background for the assay (mean PBS control value). Mann-Whitney U test was performed to compare Ab levels based on maternal IGRA status at each timepoint and paired t test to compare the same individual from 12 to 44 weeks. Benjamini-Hochberg correction was used to adjust for multiple testing. An asterisk denotes a significant difference post Benjamini-Hochberg correction with an FWER=0.05. Part of whole graphs showing classification of IgG responses into detectable and background antibody levels in infants at 12 weeks **(C)** and 44 weeks **(D)**.

Next, we measured *Mtb-*specific Ab levels in plasma from infants of mothers enrolled in the TB-Apprise cohort. No significant differences were detected for *Mtb*- or viral antigen specific antibodies based on maternal IGRA status at either timepoint in the infants (**Figures 5A, B**). High levels of IgG specific for GP120 and HA were detected in infants at 12 weeks (**Figure 1B**). Levels of *Mtb*-specific IgG were 1–2 logs lower than viral IgG in infants at 12 weeks. In contrast, at 44 weeks the presence of GP120- and HA- specific IgG dropped to antibody negative levels in most infants, while *Mtb*-specific IgG showed significant increases with age in plasma (**Figure 5B**). Additionally, we classified IgG levels as positive or negative for each antigen in the same manner as the maternal antibody data to show the increase in proportions of infants with positive *Mtb*-specific IgG and reduced number of infants with HIV and HA-specific IgG as they aged (**Figures 5C, D**). Ebola- specific IgG was also assessed at 12 and 44 weeks. Ebola- specific IgG increased from 12 to 44 weeks. However, when the MFI of Ebola IgG was compared to ESAT6/CFP10 IgG, the MFI of ESAT6/CFP10 IgG was significantly higher (**Supplementary Figures 3A, B**).

**Figure 6:**
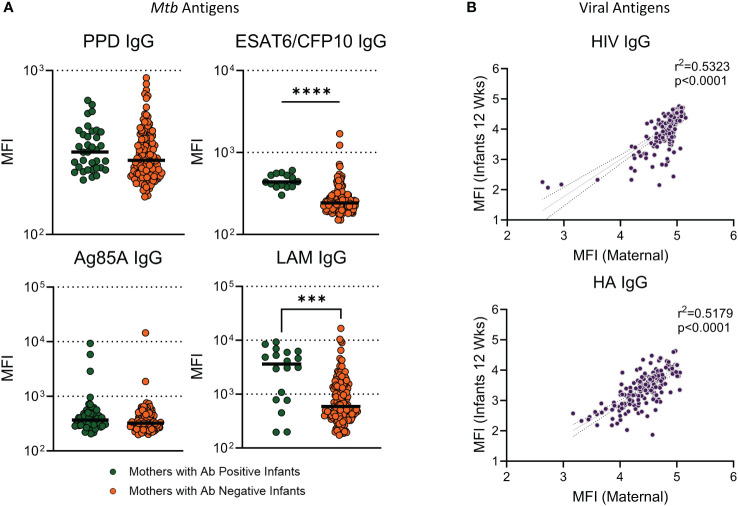
Infants with Ab positive responses to ESAT6/CFP10 and LAM had mothers with significantly higher antibody than mothers who had infants with Ab negative responses. Dot plots with median showing a comparison of **(A)** Mothers from infants with positive antibody responses versus mothers from infants with negative antibody responses to *Mtb* antigens. Mann Whitney U test was used to assess significant changes in mothers based on infant ab positivity. Significant values are represented by asterisks *p ≤ 0.05, **p ≤ 0.01, ***p ≤ 0.001, ****p ≤ 0.0001. Linear regression analysis was performed against maternal IgG levels at study entry and Infant IgG at 12 weeks for viral antigens **(B)**.

To assess the relationship between maternal antibody responses and infant antibody responses to *Mtb* antigens, mothers were grouped based on infant Ab positivity to *Mtb* antigens and assessed for significant differences. Mothers to infants with a positive antibody response to ESAT6/CFP10 and LAM had significantly higher antibody than women who had children with antibody negative responses (**Figure 6A**). There was no significant difference for PPD or Ag85A IgG. Linear regression was performed in mother/infant pairs to assess associations between maternal and infant antibody responses to HIV and HA. We found a significant positive association between levels of virus-specific IgG (HIV and HA) between mothers during pregnancy and infants at 12 weeks (**Figure 6B**).

### IGRA+ infants have reduced LAM-specific antibodies

**Figure 7:**
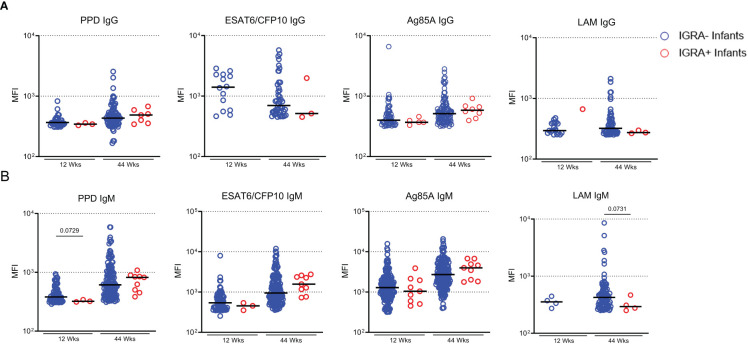
IGRA+ infants exhibit low Ab development to LAM. Dot plots with median showing a comparison of *Mtb*-specific IgG **(A)** and IgM **(B)** in Infant plasma at 12 and 44 weeks of age. Only positive Ab responses are shown for each group. Mann-Whitney U test was performed to compare Ab levels based on infant IGRA status at each timepoint and p values less than 0.1 are shown on the plot as trends.

We next sought to determine if maternally transferred or developing *Mtb*-specific antibodies were associated with protection from TB in infants. In our sub-cohort of participants from TB-APPRISE, 9 infants tested positive for IGRA at the 44-week follow-up. Comparison of positive Ab responses between IGRA+ and IGRA- infants at 44 weeks did not show a difference in proportion of positive responses (not shown) nor magnitude (MFI) of IgG or IgM levels (**Figures 7A, B**). Ab levels in these 9 individuals were assessed at the time of IGRA testing and earlier to determine any predictive associations with LTBI acquisition. We did note a trend for lower MFI of IgM against PPD at 12 weeks and LAM at 44 weeks in the IGRA+ infants compared to IGRA- infants.

## Discussion

In this study, we assessed the role of antibodies in maternal and infant responses to TB and HIV. The findings in this study suggest that maternal IGRA status does not have a significant impact on Ab levels in mothers or their children. However, we show that infants that become IGRA+ may have reduced *Mtb*-specific antibodies compared to IGRA- infants. We also explored antibody profiles in a small group of mothers who progressed to ATB. This group demonstrated altered antibody profiles at entry compared to the main cohort providing evidence to support further investigations into antibody profiles in progressors during pregnancy.

We found a strong association between HIV- and *Mtb*- specific IgG and IgM in mothers, indicating a strong response to several TB antigens. HIV and *Mtb*- antibodies also correlated to some clinical parameters such as maternal CD4 and infant weight. This suggests that there are no differences in antibody responses based on IGRA status alone amongst mothers in the cohort. A study by Lu et al. evaluated differences in *Mtb*- specific antibodies in IGRA+ people and IGRA- resisters, people with chronic exposure to *Mtb* who never progressed to an IGRA+ status and they observed no differences in antibody levels (25). This may indicate that the presence of antibody alone may be insufficient to distinguish people and their *Mtb* exposure. However, this may not be an accurate representation. The IGRA assay used in this study was the QuantiFERON-TB Gold In-Tube assay (QFTGIT), a whole blood-based ELISA assay that evaluates IFNγ secretion in response to *M. tuberculosis* peptides (ESAT-6, CFP10, and TB7.7) (26). Studies have consistently shown that the sensitivity and specificity of the QFTGIT is reduced in HIV-positive individuals, particularly in individuals with CD4 counts below 200 cells/mm^3^ (27, 28); furthermore, pregnancy induces a suppressive or highly modulated immune state which further compromises the accuracy of the QFTGIT (29). One study, utilizing samples from the P1078 clinical trial found that 68 (24%) of QGIT-positive women reverted to a QGIT-negative or intermediate status with 42 returning to a QGIT-positive status after birth. This observation was attributed to decreased IFNg responses during pregnancy (30). Another study enrolled IGRA+ women during pregnancy and found a decrease in IGRA positivity at delivery which increased postpartum. They also found greater discrepancies between the IGRA and TST results in women with HIV (31). Without a test that can accurately diagnose latent TB in these populations, it is difficult to draw finite conclusions about the impact of LTBI on antibody responses.

Women who developed ATB were compared from entry to diagnosis revealing a significant increase in *Mtb*-specific IgG, IgM and IgA1 upon diagnosis and showing the induction of a broad antibody response to *Mtb*, as previously shown (32, 33). However, when progressors were compared at study entry to non-progressors, the data showed a reduction in antibodies in circulation against ESAT6/CFP10 and LAM that preceded the progression to ATB in these individuals. ESAT6/CFP10 is a protein secreted by *Mtb* and induces a strong IFNg response. One study found that ESAT6/CFP10 was capable of inducing STAT3 which has been associated with TB progression, suggesting that women who had insufficient antibody responses to ESAT6/CFP10 may be at greater risk for progression (34, 35). LAM is a component of the cell wall of mycobacteria (including BCG and *Mtb)* and is released by metabolically active bacteria. LAM detection in urine or sputum has been investigated as a potential diagnostic tool with some success (36, 37). A recent study in a cynomolgus macaque model also showed that IgA to LAM was significantly reduced in animals with ATB compared to animals with LTBI (38). Furthermore, another study showed that children with disseminated TB had reduced LAM IgG compared to children with localized TB (39). A reduction in antibody response to ESAT6/CFP10 and LAM may suggest that progressors have an insufficiently primed immune response to *Mtb* that puts them at greater risk for progression to active TB.

Our data also revealed another potentially predictive marker for ATB progression in increased levels of Ag85A-specific IgG. The levels of these antibodies were strikingly high in a few of the ATB progressors at entry and did not increase at the time of diagnosis. Ag85A is a mycolyl transferase and is involved in lipid body formation in many mycobacterial species. As an exported enzymatic complex, Ag85A has the potential to be immunogenic and therefore elevated antibodies against Ag85A could reflect an increase in *Mtb* activity and act as a predictor of LTBI to ATB progression. In line with this, a study evaluating the use of *Mtb*-specific antibodies as a diagnostic tool to distinguish LTBI from ATB found that people with ATB had a significant increase in IgG reactivity to Ag85A proteins when compared to people with LTBI or uninfected individuals (40). Despite Ag85A antibodies being found in abundance in maternal and infant (44 wk) samples, they were not shown to be protective in any way against TB acquisition or ATB progression so it is unlikely that they can offer neutralizing activity against *Mtb*. Infant antibody responses had highly significant differences from 12 to 44 weeks with viral responses decreasing over time and *Mtb*-specific antibody responses increasing. IgG is transferred from mothers to infants during pregnancy via the neonatal Fc receptor (FcRn) (41). IgG that is transferred is highest at birth and then wanes over the course of about 6 months. Our analysis showed this decrease in HIV- and Influenza-specific IgG only. This is likely due to the strong antibody responses found in the mothers that are then passed to the infants. We confirmed the strong relationship between maternal and infant viral antibodies at 12 weeks. In contrast, *Mtb*-specific IgG increased in infants from 12 to 44 weeks. Since mothers had lower TB-specific IgG responses, it is likely that they passed fewer antibodies to infants in comparison to viral antibodies. Our data did not show a significant difference in infant Ab positivity to PPD or Ag85A based on maternal antibody level. This suggests that not all *Mtb*-specific antibodies are readily transferred from mother to infant, potentially due to maternal HIV status (42). Interestingly, ESAT6/CFP10 IgG and LAM IgG were the only *Mtb*-specific antibodies that correlated between mothers and infants at 12 weeks. Since not all the maternal antibodies correlated to the infants at 12 weeks, the increase in *Mtb*-specific IgG is most likely due to the infants developing an antibody response to these antigens potentially due to BCG vaccination or exposure to environmental TB. Ebola- specific IgG was also included in our analysis and we saw an increase from 12 to 44 weeks which is likely a result of IgG levels beginning to rise in infants. However, when compared to the MFI of ESAT6/CFP10 IgG, we found that the MFI of ESAT6/CFP10 IgG was significantly higher than the MFI of Ebola IgG at all time points. This confirms that the increase in *Mtb*- specific IgG is due to environmental exposure to *Mtb*. Due to the presence of antibodies against ESAT6/CFP10, an antigen unique to TB, it is likely that the responses we see in some of the infants are due to environmental TB. Lubyayi et al. previously demonstrated that infants from mothers who were either IGRA+ or IGRA- had both undergone sensitization to TB antigen, ignoring maternal IGRA status. However, they determined that the infants’ immune response to BCG was not hindered (17).

Since LAM-specific IgG, IgM, and IgA1 were significantly increased in the LTBI group compared to progressors we next questioned whether this provided evidence of protection or risk for TB acquisition in infants. We further suspected that LAM IgG may be an indicator of protection or risk due to the correlation between mothers and infants at 12 weeks. The number of infants that tested positive for IGRA at 44 weeks was small in relation to the main cohort, so we did not find any significant differences between antibody responses in IGRA- and IGRA+ infants, however we observed trends for reduced PPD IgM (p=0.0729) and LAM IgM (p=0.0731) in the IGRA+ infants. Due to the small sample size, we cannot confirm whether the amount of LAM IgG that is passed from mother to infant is protective. However, future studies with larger sample sizes may be able to address this question. Furthermore, this data does not rule out differences in antibody function. Studies have shown that maternal HIV infection can alter the transfer of functional antibodies (22, 43). It will be important to follow these experiments up with functional antibody analysis to determine whether there is any impact on the function of the antibodies that are transferred or that develop with age.

This study evaluated a unique group of mother-infant pairs which provided insight into *Mtb*, HIV, and HA-specific antibody responses. However, the limitations of our study include small sample size in exploratory analysis and a lack of functional profiling of antibodies. There were only 5 women who progressed to ATB, reducing the overall power of exploratory analysis of progressors. While the findings related to progression to TB reflected those of other studies, larger studies evaluating progression post pregnancy are needed to corroborate the findings from this study. Similarly, only a few infants became IGRA+ at 44 weeks (n=9). While some trends were observed, the power of this analysis was likely reduced. To better understand potential factors affecting the risk of ATB acquisition in infants, larger studies are needed to provide sufficient power. Finally, this study only assesses antibody epitope and isotype profiles and does not evaluate the functional profile of the antibodies that were detected. While there may not be any difference in the amount of antibodies that were present, this does not rule out potential changes in antibody functional profiles based on maternal IGRA status. More studies evaluating functional profiles in mothers and infants will provide greater insight into whether antibody profiles are altered by maternal IGRA status and whether this may impact the infants’ response to BCG.

The critical characteristics of protective immunity to tuberculosis are unknown and underpin the failure to implement an effective vaccine beyond the current BCG vaccination approach. The humoral response to BCG vaccination is poorly defined in neonates, despite this being the recommended age for vaccine administration. BCG vaccination studies in humans have demonstrated increases in PPD-specific IgG in serum 12 weeks after vaccination; however, these studies have been largely limited to adults (44). Research surrounding the immune response to TB has focused on the CD4 T cell response and its role in controlling the infection in macrophages. This has left researchers with the impression that antibodies play little to no role in the control of infection or in response to BCG. Studies that have investigated the humoral response have revealed an association between TB-specific IgG responses and protection against active and disseminated TB (45). Our data suggests that maternal IGRA does not impact maternal or infant antibody responses to *Mtb* antigens, suggesting that infant responses to BCG are not likely to be impaired due to antibody epitope and isotype profile alone. The combined analysis between mothers and infants also shows evidence of potential biomarkers for further study in progressors in pregnancy. We have also identified potential factors that could contribute to protection or risk of acquiring ATB in infants. Together our data demonstrates potential changes in humoral profiles due to TB infection that may provide a basis for using LAM IgG as a biomarker for progression and as a potential risk factor for infants with insufficient IgG from mothers. Further studies are needed to fully understand the impact that reduced LAM IgG has on mothers and their infants and to further corroborate these findings.

## Data availability statement

The original contributions presented in the study are included in the article/**Supplementary Material**. Further inquiries can be directed to the corresponding author.

## Ethics statement

The studies involving humans were approved by International Maternal Pediatric Adolescent AIDS Clinical Trials Network. The studies were conducted in accordance with the local legislation and institutional requirements. The human samples used in this study were acquired from the International Maternal Pediatric Adolescent AIDS Clinical Trials Network (IMPAACT) P1078/TB APPRISE cohort (clinical trial NCT01494038). Written informed consent to participate in this study was provided by the participants’ legal guardian/next of kin.

## Author contributions

KH: Investigation, Methodology, Software, Visualization, Writing – original draft, Writing – review & editing, Formal analysis. LD: Conceptualization, Data curation, Supervision, Writing – review & editing, Visualization. EG: Writing – review & editing, Formal analysis. SuP: Conceptualization, Writing – review & editing. JM: Writing – review & editing. GM: Conceptualization, Data curation, Writing – review & editing. AG: Conceptualization, Writing – review & editing. SaP: Conceptualization, Funding acquisition, Resources, Supervision, Writing – review & editing.

## Supplementary material

Supplementary Figure 1 Ebola- specific Antibodies in mothers. Dot plots with median bars showing a comparison of Ebola- specific IgG **(A)**, IgM **(B)**, and IgA **(C)** between IGRA- and IGRA+ mothers. Dot plots with median bars showing a comparison of Ebola- specific IgG to Mtb- specific IgG **(D)**. Green dotted lines represent the background for the assay (mean PBS control value). Mann-Whitney U test was performed to compare Ab levels based on maternal IGRA and to compare Ebola IgG to Mtb- specific IgG. Significant values are represented by asterisks *p ≤ 0.05, **p ≤ 0.01, ***p ≤ 0.001, ****p ≤ 0.0001.

Supplementary Figure 2
*HIV-, HA*-, and Ebola- specific antibodies do not change in maternal plasma with active TB diagnosis post-delivery. Dot plots comparing levels of HIV- **(A)**, HA- **(B)**, and Ebola- specific IgG **(C)** in active TB progressors at study entry and the time of diagnosis (n=5). Blue dotted lines represent the background for the assay (mean PBS control value). Active TB progressors were compared from entry to diagnosis using Wilcoxon Sign Rank test. Significant values are represented by asterisks *p ≤ 0.05, **p ≤ 0.01, ***p ≤ 0.001, ****p ≤ 0.0001.

Supplementary Figure 3 Changes in Ebola- specific IgG in infants at 12 and 44 weeks. Dot plots with median bars showing a comparison of Ebola- specific **(A)** IgG in infant plasma at 12 and 44 weeks of age. Dot plots with median bars showing a comparison of Ebola- specific and ESAT6/CFP10- specific IgG at 12 weeks **(A)** and 44 weeks **(B)**. Green dotted lines represent the background for the assay (mean PBS control value). A paired t test was used to compare the same individual from 12 to 44 weeks. The MFI of Ebola- specific and ESAT6/CFP10- specific IgG were also compared in each group at 12 and 44 weeks using a Kruskal- Wallis test and corrected for multiple testing. Significant values are represented by asterisks *p ≤ 0.05, **p ≤ 0.01, ***p ≤ 0.001, ****p ≤ 0.0001.
